# Age- and Sex-Dependent Changes in Locus Coeruleus Physiology and Anxiety-Like Behavior Following Acute Stressor Exposure

**DOI:** 10.3389/fnbeh.2022.808590

**Published:** 2022-02-25

**Authors:** Olga Borodovitsyna, John A. Tkaczynski, Claire M. Corbett, Jessica A. Loweth, Daniel J. Chandler

**Affiliations:** ^1^Graduate School of Biomedical Sciences, Rowan University, Stratford, NJ, United States; ^2^Department of Cell Biology and Neuroscience, Rowan University School of Osteopathic Medicine, Stratford, NJ, United States

**Keywords:** sex differece, adolescence, adulthood, stress, locus coeruelus

## Abstract

Adolescence is a critical period of development with increased sensitivity toward psychological stressors. Many psychiatric conditions emerge during adolescence and animal studies have shown that that acute stress has long-term effects on hypothalamic-pituitary-adrenal axis function and behavior. We recently demonstrated that acute stress produces long-term electrophysiological changes in locus coeruleus and long-lasting anxiety-like behavior in adolescent male rats. Based on prior reports of increased stress sensitivity during adolescence and increased sensitivity of female locus coeruleus toward corticotropin releasing factor, we hypothesized that the same acute stressor would cause different behavioral and physiological responses in adolescent female and adult male and female rats one week after stressor exposure. In this study, we assessed age and sex differences in how an acute psychological stressor affects corticosterone release, anxiety-like behavior, and locus coeruleus physiology at short- and long-term intervals. All groups of animals except adult female responded to stress with elevated corticosterone levels at the acute time point. One week after stressor exposure, adolescent females showed decreased firing of locus coeruleus neurons upon current injection and increased exploratory behavior compared to controls. The results were in direct contrast to changes observed in adolescent males, which showed increased anxiety-like behavior and increased spontaneous and induced firing in locus coeruleus neurons a week after stressor exposure. Adult males and females were both behaviorally and electrophysiologically resilient to the long-term effects of acute stress. Therefore, there may be a normal developmental trajectory for locus coeruleus neurons which promotes stress resilience in adults, but stressor exposure during adolescence perturbs their function. Furthermore, while locus coeruleus neurons are more sensitive to stressor exposure during adolescence, the effect varies between adolescent males and females. These findings suggest that endocrine, behavioral, and physiological responses to stress vary among animals of different age and sex, and therefore these variables should be taken into account when selecting models and designing experiments to investigate the effects of stress. These differences in animals may also allude to age and sex differences in the prevalence of various psychiatric illnesses within the human population.

## Introduction

Adolescence is an important period of transition between childhood and adulthood and is a sensitive period for stressor exposure (van Dijken et al., 1993). Neuroendocrine development in adolescence is characterized by maturation of the central nervous system (CNS) and development of the hypothalamic-pituitary-adrenal (HPA) axis. A surge of gonadal hormones during puberty renders the adolescent HPA axis more susceptible to stress. For example, it takes almost twice as long for adolescent rats to return to the normal baseline plasma concentration of the stress hormone corticosterone during recovery after stress when compared to adults (Romeo et al., 2004a,b). Additionally, adolescence in humans is characterized by an increase in the baseline concentration of the primary stress hormone in humans, cortisol, and an increase in the stress reactivity of the HPA axis (Adam, 2006). There are notable sex differences in HPA axis function as well. For example, female rats have been shown to have higher baseline plasma corticosterone levels and increased responsiveness to stressors compared to males (Kitay, 1961). Conversely, a human study has shown that stress anticipation leads to higher levels of cortisol release in males than females (Kirschbaum et al., 1992).

Behaviorally, adolescence in humans is characterized by a pursuit of independence, changing social status, and increased risk-taking behavior. These changes are paralleled in rodents by increased play behavior and social interactions, and greater exploration of novel environments (Spear, 2000). It has been reported that peripubertal stress leads to increased risk-taking behavior and decreased anxiety-like behavior in late adolescence (Toledo-Rodriguez and Sandi, 2011). Increased behavioral and emotional susceptibility to stress and risk-taking behavior in adolescence could be explained in part by faster development of limbic structures, including the central nucleus of the amygdala, compared to prefrontal cortex (PFC) (Arain et al., 2013). Because there have been inconsistent reports of the effects of stress on behavior, cognitive function, and HPA axis responsiveness (McCormick et al., 2010), additional research is necessary to compare how the effects of stress vary according to an animal’s age and sex.

A major brain nucleus involved in the modulation of the central stress response is the locus coeruleus (LC), which exerts its actions through the release of the neuromodulator norepinephrine (NE). It is activated by stress through release of corticotropin releasing factor (CRF), which interacts with corticotropin releasing factor receptor 1 (CRFR1). This increases tonic firing of LC neurons and promotes forebrain release of NE (Valentino et al., 1983, 1992; Jedema and Grace, 2004). While there is an established relationship between LC discharge rates and behavioral indices of arousal and anxiety-like behavior (Poe et al., 2020), the long-term effects of stress on LC, and its relationship to behavior, is less clear. The transition from adolescence into adulthood is characterized by a number of changes in the LC/NE system: decreased NE transporter content in PFC (Bradshaw et al., 2016), age-related decline in LC spontaneous cell firing (Olpe and Steinmann, 1982), and increased capacity for the recovery and adaptation in response to social stress (Zitnik et al., 2016). Accordingly, it is worthwhile to investigate if LC might mediate chronic changes in both physiology and behavior induced by an acute stressor. Indeed, studies have shown that behavioral and physiological changes occur in adulthood after adolescent stress (Yohn and Blendy, 2017). Postnatal environmental changes are also known to impact the developing brain (Kolb and Gibb, 2011), and, thus, stressor exposure in adolescence may chronically alter LC in such a way that changes behavior in later life.

We have previously shown that a single acute stressor exposure (15 min combined predator odor and physical restraint) is sufficient to produce behavioral and LC electrophysiological changes a week after stress in adolescent male rats (Borodovitsyna et al., 2018a,^2020^). However, this study included only male rats, and it is known that the LC is sexually dimorphic in both morphology and stress responsiveness (Bangasser et al., 2016). Specifically, there is evidence for denser afferentation from limbic areas in female LC, prolonged activity of CRF receptor activation on LC neurons as a result of slower receptor internalization, and presynaptic modulation of NE release with increased estrogen-dependent release and decreased degradation of NE (Bangasser et al., 2016). Sex-differential expression of more than 100 genes has also been identified in adult mouse LC (Mulvey et al., 2018). Based on these findings, a more comprehensive analysis of how age, sex, and stress interact to modulate anxiety-like behavior is necessary. Therefore, in this study, both adolescent and adult male and female rats are included. Here we describe how electrophysiological properties of LC neurons and anxiety-like behavior are affected by stressor exposure in multiple assays at different time points. We further assess the HPA axis response based on serum corticosterone levels. Together, endocrine, physiological, and behavioral responses to stressor exposure among animals of both sexes and different ages will help to create a more refined picture of the effects of acute stressor exposure on the LC/NE system.

## Materials and Methods

### Subjects

Adolescent (30–35 PND) male and female, and adult (77–82 PND) virgin male and female Sprague Dawley rats (Taconic Farms, Germantown, NY, United States), were housed two to three per cage on a 12 h reverse light schedule (lights on at 9:00 pm) with access to standard rat chow and water *ad libitum*. Animals were handled prior to and during experimentation to habituate to the researcher. Animal protocols were approved by the Rowan University Institutional Animal Care and Use Committee and were conducted in accordance with National Institutes of Health *Guide for the Care and Use of Laboratory Animals.*

### Stressor Exposure

Rats underwent stessor exposure or control conditions as previously described (Borodovitsyna et al., 2018a,^2020^). Briefly, rats were placed in a rodent restrainer (Harvard Apparatus), which was then placed inside of a plastic bell chamber. Predator odor (2,4,5-trimethylthiazole, TMT; Sigma-Aldrich, St. Louis, MO, United States) was delivered to the chamber by pipetting 100 μL TMT onto a piece of filter paper and placing it within a plastic tube connected by silicon tubing on one end to the chamber and the other end to an air supply. Turning on the air supply caused the odor to be carried to the chamber continuously during the 15 min of restraint. Control animals were placed in an identical bell chamber for fifteen minutes, but they were not restrained and no odor was delivered.

### Serum Corticosterone

In some of the animals used for behavioral and electrophysiological experiments, blood (0.2–0.4 mL) was collected from the saphenous vein immediately before the onset of stress or control conditions (labeled as “baseline”) and 35 min after the onset (labeled as “35 min”). In subjects who were studied for long-term effects, in addition to the baseline and acute collections, blood was also collected from the saphenous vein after behavioral testing one week after control or stress conditions and was labeled as “1 week”. In all cases, blood was collected per the protocol for frequent blood collection from the lateral saphenous vein in unanesthetized animals (Beeton et al., 2007; Parasuraman et al., 2010) into 1.5 mL Eppendorf tubes and left for at least 10 min to coagulate, and then centrifuged at 12,800 rcf for 3 min. Serum was kept at –80°C prior to analysis using a Corticosterone Enzyme Immunoassay kit from Enzo (ADI-900-097). Results were calculated using Four Parameter Logistic Curve fit in R-Studio.

### Estrous Cycle Monitoring

Vaginal swabs were taken regularly from adult female rats throughout the study (4–5 days in a row followed by 2–3 days off) in order to effectively track each animal’s estrous cycle. Females were swabbed at the onset of the dark cycle (∼9 am–10 am) the weeks before electrophysiology recordings and approximately 30 min before the animals were euthanized for recordings to identify the estrous stages of the rats on the days of stressor exposure and the days of behavior. Vaginal samples were collected by gently swabbing the vaginal canal using a saline-dipped cotton-tipped swab and applying to glass microscope slides. Slides were stained with toluidine blue and examined using light microscopy. Estrous cycle stage was determined by the presence and morphology of cells based on previously published criteria by other laboratories (Hubscher et al., 2005; Cora et al., 2015). Each sample was classified as one of the following four stages: metestrus (diestrus I/D1), diestrus (diestrus II/D2), proestrus, and estrus. Metestrus was classified based on the presence of approximately equal numbers of nucleated epithelial cells, non-nucleated cornified epithelial cells, and leukocytes. Diestrus was classified based on the observation of few cells, including leukocytes and occasional epithelial cells. The proestrus phase was classified based on the presence of 75% or more of nucleated epithelial cells, and the estrus phase was classified by the presence of 75% or more of non-nucleated cornified epithelial cells. All female rats exhibited normal cycling (4–5 days cycles) throughout the study.

### Elevated Plus Maze

Immediately after exposure to stress or control conditions, rats were placed in the center of an elevated plus maze (EPM). The EPM consisted of a plus shaped black plexiglass apparatus elevated 76 cm off the ground with two sets of opposing arms (each arm = 40cm in length) meeting in a central 10 cm × 10 cm area. Two opposing arms have vertical walls extending 30 cm from the floor of the maze, while the other two arms do not have walls. Rats were allowed to explore the maze for 10 min, during which their activity was filmed with an infrared camera. At the conclusion of each test, rats were returned to their home cage for a week. Behavior was scored using AnyMaze behavioral tracking software (Stoelting, Wood Dale, IL, United States).

### Open Field Test

One week after testing in the EPM, rats were placed in the center of an open field test (OFT) apparatus. The OFT apparatus consisted of a 90 cm × 90 cm × 30 cm black plexiglass box. Rats were allowed to explore the apparatus for 10 min, during which their activity was filmed with an infrared camera situated above the maze. At the conclusion of each test, rats were sacrificed for electrophysiological recordings. Behavior was scored using AnyMaze behavioral tracking software (Stoelting, Wood Dale, IL, United States). Rats were tested in different mazes at the two time points to eliminate the possibility of habituation to any one test confounding anxiety-like behavior.

### Brain Slice Preparation

Acute brain slices were prepared as previously described (Borodovitsyna et al., 2018a,^2020^). Briefly, rats were deeply anesthetized with an intraperitoneal injection of Euthasol (100 mg/kg, Virbac, St. Louis, MO, United States) and transcardially perfused with ice cold oxygenated sucrose artificial cerebrospinal fluid (aCSF) of the following composition, in mM: sucrose 58.4, NaCl 85, KCl 2.5, CaCl_2_ 2.4, NaH_2_PO_4_ 1.2, MgCl_2_ 1.3, NaHCO_3_, D-glucose 25. Rats were then rapidly decapitated and the skull was removed so that gross coronal cuts could be made at the level of the medulla and the pineal gland. The blocked brain was submerged in the sucrose-aCSF for 1–2 min after which it was transferred to a piece of filter paper. The dorsal aspect of the brain was cut at 200 μM thick horizontal sections using Compresstome VF-300-0Z tissue slicer. Sections containing LC were transferred into aCSF of the following composition, in mM: NaCl 126, KCl 2.5, CaCl_2_ 2.4, NaH_2_PO_4_ 1.2, MgCl_2_ 1.3, NaHCO_3_ 25, D-glucose 11 and continuously bubbled with 95% O_2_/5% CO_2_ and maintained at 36.0°C. After 1 h, the holding incubator was maintained at room temperature.

### Electrophysiological Recordings

Slices were individually transferred to a recording chamber which was continuously superfused with oxygenated aCSF and maintained at 37°C by a Warner Instrument Corporation in-line heater (model 60-01013). LC was visualized as a semi-translucent, crescent-shaped region located lateral to the fourth ventricle at 10× magnification using an Olympus BX51WI fixed-stage upright microscope with differential interference contrast and an infrared filter. Individual LC neurons were visualized with a 40× immersion lens and QImaging Rolera Bolt camera using QCapture Pro software. Neurons were approached with sharp glass electrodes (resistance = 5–10 MΩ) controlled with Sutter MPC-200 manipulators. Electrodes were filled with intracellular solution of the following composition, in mM: KCl 20, K-gluconate 120, MgCl_2_ 2, EGTA 0.2, HEPES 10, Na_2_ATP 2, and 0.15% biocytin. After a GΩ seal was established between the pipette and neuronal membrane, the membrane was ruptured and whole-cell recordings were obtained. To assess membrane properties in current clamp mode, spontaneous activity was recorded for 60 s after stabilization of cell firing and the average firing rate was calculated. Cells were then subjected to a series of increasing current steps from -250 to 300 pA with 50 pA intervals between sweeps, and the input resistance and number of action potentials fired in response to each level of current was determined. General exclusion criteria for recordings were input resistance <100 MΩ during evoked firing and/or more than 6 Hz spontaneous firing. Otherwise both spontaneously active and “silent” neurons were included.

### Data Analysis

Electrophysiological data were recorded using Molecular Devices ClampEx 10.2 and MultiClamp 700B acquisition software and analyzed with ClampFit 10.2. Behavioral data were acquired and analyzed using AnyMaze behavioral tracking software (Stoelting, Wood Dale, IL, United States). Statistical analyses were performed with GraphPad Prism Version 8.0.1. Data was tested for normality using Shapiro–Wilk test. Normally distributed data were tested for equal variances of distribution, and, for the data with equal variances, two-tailed *t*-test was performed. If variances were unequal, a Welch modification of the *t*-test was conducted. Wilcoxon Rank Sum test (Mann–Whitney U test) was used for non-normally distributed data sets. Within each group (adolescent male, adolescent female, adult male, adult female) a 2 (treatment; control vs sex) × 2 (time; baseline vs 35 min vs 1 week) ANOVA was used to assess for differences in corticosterone concentrations. Planned comparisons with Bonferroni corrections applied to control for multiple contrasts between the following groups were used: (1) control: baseline vs 35 min; (2) control: baseline vs 1 week; (3) stress: baseline vs 35 min; (4) stress: baseline vs 1 week; (5) 35 min: control vs stress; and (6) 1 week: control vs stress. For adolescent males, tests of the significance of the Pearson correlation coefficient were used to explore relationships between various behavioral and physiological parameters. Statistical significance was set to alpha = 0.05 in all analyses. Error bars in all figures are presented as mean ± SEM.

## Results

### Adolescent Females

**FIGURE 1:**
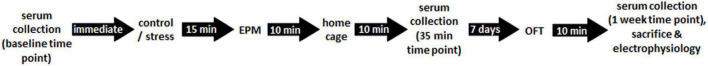
Experimental timeline for all groups. Rats are bled from the lateral saphenous vein to collect serum for corticosterone quantification and are then immediately exposed to control or stress conditions for 15 min. Anxiety-like behavior is then immediately tested in the elevated plus maze for 10 min, and animals are then returned to home cages. Ten minutes later (35 min after the start or control conditions) blood is collected again from the lateral saphenous vein. Rats are then housed undisturbed for a week, at which point anxiety-like behavior is tested again in the open field test. At the conclusion of the test, blood is again collected from the lateral saphenous vein and animals are sacrificed for electrophysiological recordings.

**FIGURE 2:**
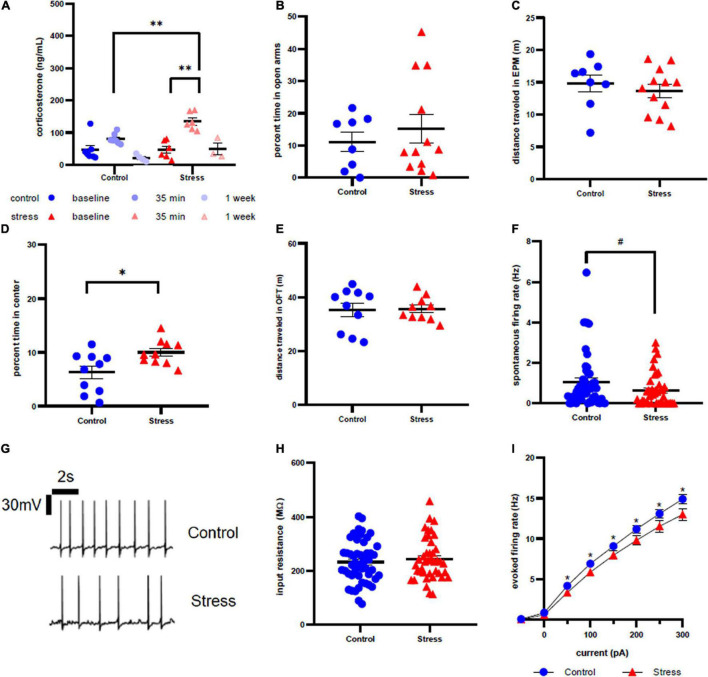
Stress response in adolescent female rats. **(A)** Serum corticosterone concentration is higher 35 min after stress compared to baseline and to control at the same time point. **(B)** There is no effect of stress on time spent in open arms of the elevated plus maze immediately after stress or control conditions. **(C)** There is no effect of stress on distance traveled in the EPM. **(D)** Animals spend more time in the center of the open field test one week after stress compared to control. **(E)** There is no effect of stress on distance traveled in the OFT one week after stress. **(F)** There is no effect of stress on spontaneous firing rate of LC neurons one week after stress. **(G)** Representative traces for spontaneous firing in control **(top)** and stress **(bottom)** groups. **(H)** There is no effect of stress on input resistance of LC neurons one week after stress. **(I)** Stress decreases the excitability of LC neurons one week after stress. ^#^*p* < 0.1; ^∗^*p* < 0.05; ^∗∗^*p* < 0.01.

To demonstrate the effects of acute stress on adolescent female rats, the study was performed according to the experimental timeline shown in Figure 1. Serum corticosterone levels from female rats are shown in Figure 2A. Acute exposure to TMT and restraint stress significantly increased corticosterone levels 35 min after the beginning of stress exposure compared to control animals (*t* = 4.409, df = 11, *p* = 0.0060), along with a significant difference between baseline and 35 min time points exclusively in the stress group (*t* = 5.548, df = 10, *p* = 0.0012).

Anxiety-like behavior was assessed immediately after (Figure 2B) and one week after (Figure 2D) stress in the EPM and OFT, respectively. Time spent in the open arms and time spent in the center of open field were used as measurements of anxiety-like behavior. We did not observe any statistical difference in time spent in the open arms in the EPM between control and stress rats (*t* = 0.7003, df = 18, *p* = 0.4927). However, stressed female rats spent significantly more time in the center of the OFT a week after stress (*t* = 2.691, df = 18, *p* = 0.0149). There was also no effect of stress on distance traveled in the EPM immediately after stress (Figure 2C, *t* = 0.5167, df = 18, *p* = 0.5167) or in the OFT a week after stress (Figure 2E, *t* = 0.1058, df = 18, *p* = 0.9169).

There was a non-significant trend toward a decrease in spontaneous firing rate of female LC neurons one week after stressor exposure compared to controls (*p* = 0.0819, U = 807.5; Figure 2F), with representative traces shown in figure 2G. Input resistance did not significantly differ between stress and control groups (*t* = 0.6814, df = 89, *p* = 0.4974, Figure 2H). However, stressor exposure significantly decreased the LC neuronal firing rate in response to current injection for all current steps beyond 0 pA (Figure 2I): 50 pA (*U* = 782.5, *p* = 0.0360), 100 pA (*U* = 738, *p* = 0.0143), 150 pA (*U* = 787.5, *p* = 0.0410), 200 pA (*U* = 763, *p* = 0.0252), 250 pA (*U* = 748, *p* = 0.0184), 300 pA (*U* = 723, *p* = 0.0105).

### Adolescent Males

**FIGURE 3:**
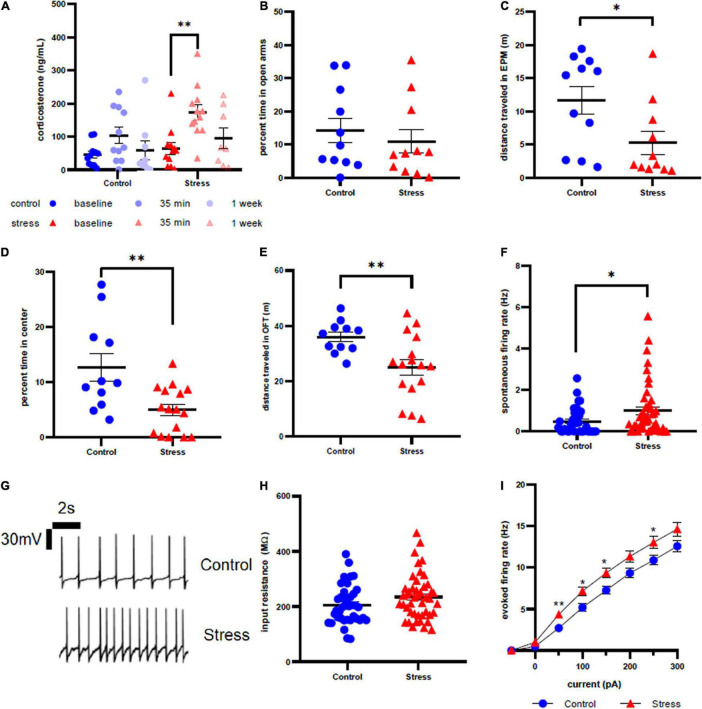
Stress response in adolescent male rats. **(A)** Serum corticosterone concentration is higher 35 min after stress compared to baseline. **(B)** There is no effect of stress on time spent in open arms of the elevated plus maze immediately after stress or control conditions. **(C)** Stress decreases distance traveled in the elevated plus maze immediately after stress or control conditions. **(D)** Animals spend less time in the center of the open field test one week after stress compared to control. **(E)** Prior stress decreases distance traveled in the open field test. **(F)** Stressor exposure increases the spontaneous firing rate of LC neurons one week after stress. **(G)** Representative traces for spontaneous firing in control **(top)** and stress **(bottom)** groups. **(H)** There is no effect of stress on input resistance of LC neurons one week after stress. **(I)** Stress increases the excitability of LC neurons one week after stress. ^∗^*p* < 0.05; ^∗∗^*p* < 0.01.

Adolescent male rats were subject to the same experimental timeline as adolescent female rats (Figure 1). Corticosterone values were significantly higher in stressed animals at the 35 min time point compared to baseline (*U* = 20, *p* = 0.0018, Figure 3A). There were no other significant differences between groups.

Although we did not observe a difference in percent open arm time in the EPM between control and stressed adolescent males (*U* = 52, *p* = 0.6063, Figure 3B), stressor exposure significantly decreased total distance traveled in the EPM (*U* = 27, *p* = 0.0281, Figure 3C). One week later, stressed animals also spent less time in the center of the OFT compared to controls (*t* = 3.189, df = 25, *p* = 0.0038, Figure 3D), along with significantly less distance traveled (*t* = 3.223, df = 23.29, *p* = 0.0037, Figure 3E). Consistent with our previously published results, LC neurons from stressed adolescent male rats had a significantly higher spontaneous firing rate than those from control animals (*U* = 620, *p* = 0.0300, Figure 3F). Representative traces are shown in Figure 3G. However, input resistance did not differ between stress and control groups (*U* = 671, *p* = 0.0931, Figure 3H). In contrast to adolescent females, the firing rate in response to current injection was significantly higher in LC neurons from stressed adolescent males than controls at 50 pA (*U* = 558.5, *p* = 0.0057), 100 pA (*U* = 586.5, *p* = 0.0129), 150 pA (*U* = 590.5, *p* = 0.0146) and 250 pA current injection (*U* = 624.5, *p* = 0.0481; Figure 3I).

### Adult Females

**FIGURE 4:**
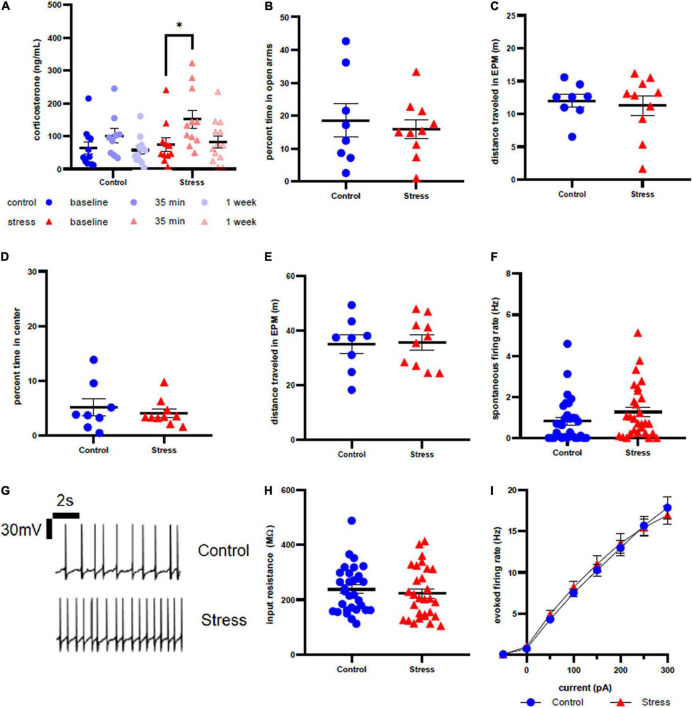
Stress response in adult female rats. **(A)** Serum corticosterone concentration is higher 35 min after stress compared to baseline. **(B)** There is no effect of stress on time spent in open arms of the elevated plus maze immediately after stress or control conditions. **(C)** There is no effect of stress on distance traveled in the elevated plus maze immediately after stress or control conditions. **(D)** There is no effect of stress on time spent in the center of the open field test one week after stress. **(E)** There is no effect of stress on distance traveled in the open field test one week after stress. **(F)** There is no effect of stress on spontaneous firing rate of LC neurons one week after stress. **(G)** Representative traces for spontaneous firing in control **(top)** and stress **(bottom)** groups. **(H)** There is no effect of stress on input resistance of LC neurons one week after stress. **(I)** There is no effect of stress on the excitability of LC neurons one week after stress. ^∗^*p* < 0.05.

Adult female rats were subject to the same experimental timeline as the other groups (Figure 1). As with the other groups, there was a significant difference between the baseline and 35 min time points in the stress-exposed group (*U* = 20, *p* = 0.0384, Figure 4A). There were no other significant differences in the other comparisons. Most of the animals were in the diesterus phase of the cycle at the moment of both stress (16 out of 18 rats) and sacrifice (13 out of 18 rats).

Anxiety-like behavior of adult female rats was unaffected by stressor exposure in both the EPM at the acute time point (*t* = 0.4749, df = 16, *p* = 0.6413, Figure 4B) and the OFT at the 1 week time point (*U* = 36, *p* = 0.7618, Figure 4D). There was no difference in distance traveled in the EPM immediately after (*t* = 0.1519, df = 14, *p* = 0.8814, Figure 4C) or in the OFT 1 week after stress or control conditions (*t* = 0.1314, df = 16, *p* = 0.8971, Figure 4E). Stressor exposure also did not significantly affect spontaneous firing rate (*U* = 333.5, *p* = 0.0856, Figure 4F), input resistance (*U* = 390.5, *p* = 0.3876, Figure 4H), or evoked firing rate in response to any level of current injection (Figure 4I) in LC neurons. Representative traces are shown in Figure 4G.

### Adult Males

**FIGURE 5:**
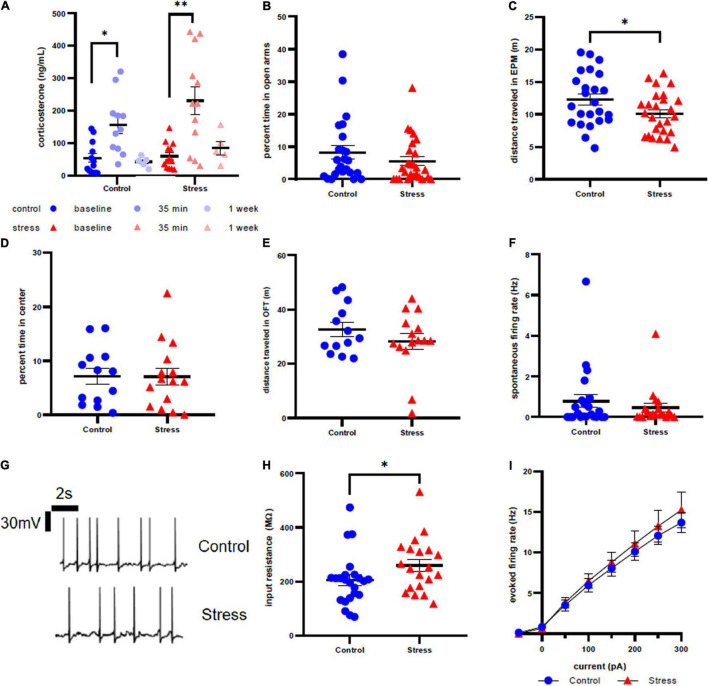
Stress response in adult male rats. **(A)** Serum corticosterone concentration is higher 35 min after stress compared to baseline and 35 min after control compared to baseline. **(B)** There is no effect of stress on time spent in open arms of the elevated plus maze immediately after stress or control conditions. **(C)** Stress decreases distance traveled in the elevated plus maze immediately after stress or control conditions. **(D)** There is no effect of stress on time in the center of the open field test one week after stress. **(E)** There is no effect of stress on distance traveled in the open field test one week after stress. **(F)** There is no effect of stress on spontaneous firing rate of LC neurons one week after stress. **(G)** Representative traces for spontaneous firing in control **(top)** and stress **(bottom)** groups. **(H)** There is a significant increase in input resistance of LC neurons one week after stress. **(I)** There is no effect of stress on the excitability of LC neurons 1 week after stress. ^∗^*p* < 0.05; ^∗∗^*p* < 0.01.

Adult male rats were subject to the same experimental timeline as the other groups (Figure 1). Corticosterone levels were significantly higher in the stressor-exposed rats at the 35 min time point relative to baseline (*t* = 5.033, df = 11.03, *p* = 0.0024, Figure 5A), but a significant difference was also seen between the baseline and 35 min time points in the control condition (*U* = 19, *p* = 0.0168, Figure 5A). No other differences were seen in the other comparisons made.

Anxiety-like behavior of adult male rats was unaffected by stressor exposure in both the EPM at the acute time point (*U* = 253, *p* = 0.2570, Figure 5B) and the OFT at the one week time point (*p* = 0.9801, *t* = 0.02524, df = 26, Figure 5D). However, we did identify a significant effect of stress on distance traveled in EPM (*t* = 2.108, df = 48, *p* = 0.0403, Figure 5C), although there was no difference in distance traveled in OFT a week after stress (*U* = 89, *p* = 0.7168, Figure 5E). Additionally, spontaneous firing rate (*U* = 211, *p* = 0.8547, Figure 5F) and evoked LC neuronal firing rates (Figure 5I) did not differ between stress or control conditions. However, there is a significantly higher input resistance in the stress group when compared to control (*U* = 149.5, *p* = 0.0499, Figure 5H). Representative traces are shown in Figure 5G.

### Correlation Analysis

**FIGURE 6:**
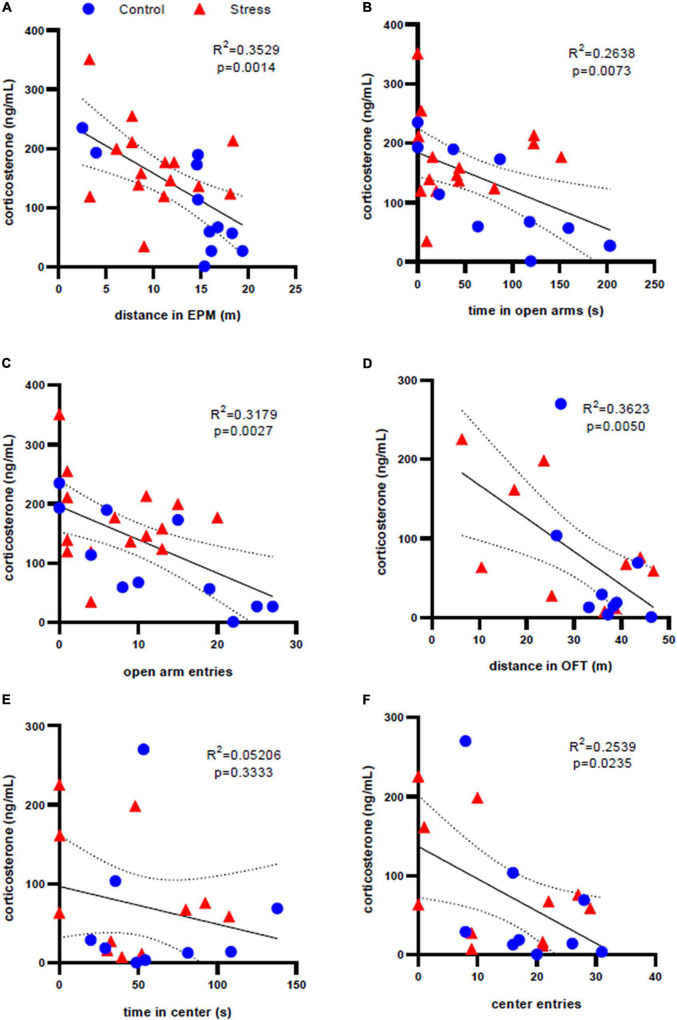
Correlation analysis of corticosterone levels and anxiety-like behavior in adolescent male rats. There is a significant correlation between serum corticosterone concentration 35 min after stress and elevated plus maze behaviors of **(A)** distance traveled, **(B)** time in the open arms, and **(C)** open arm entries. There is a significant correlation between serum corticosterone concentration one week after stress and open field test behaviors of **(D)** distance traveled and **(F)** center entries, but not **(E)** time in the center. Dotted lines represent 95% confidence interval.

Because of a clearer relationship between stressor exposure and anxiety-like behavior in adolescent male rats, we sought to explore how corticosterone levels in this group at the 35 min and one week time points related to behavior in both the EPM and OFT. Correlation mapping of data from all adolescent males (both stress and control) shows that distance traveled in the EPM (*R*^2^ = 0.3529, *p* = 0.0014, Figure 6A) and both time spent in the open arms of EPM (*R*^2^ = 0.2638, *p* = 0.0073, Figure 6B) and number of entries to the open arms (*R*^2^ = 0.3179, *p* = 0.0027, Figure 6C) are significantly negatively correlated with corticosterone levels at the 35 min time point. Similarly, considering all adolescent male rats regardless of treatment, there were significant negative correlations between corticosterone at the one week time point and distance traveled in the OFT (*R*^2^ = 0.3623, *p* = 0.0050, Figure 6D) and corticosterone at the 1 week time point and number of entries to the center of the OFT (*R*^2^ = 0.2539, *p* = 0.0235, Figure 6F). There was no correlation between corticosterone levels and time spent in the center of OFT (*R*^2^ = 0.05206, *p* = 0.3333, Figure 6E). No significant correlations between firing frequency and behavior were identified in adolescent males. No significant correlations were identified in adolescent or adult females or adult males.

## Conclusion

Previous findings from our lab have shown that a single episode of combined restraint and predator odor stress in adolescent male rats leads to specific changes in LC physiology and anxiety-like behavior (Borodovitsyna et al., 2018a,^2020^). Here, we extend these findings to show if and how the same stressor affects HPA axis activation, LC physiology, and behavior in adolescent and adult rats of both sexes.

The HPA axis is the central regulator of the stress response. Its activation results in the release of steroid hormones by the adrenal glands, which leads to energy metabolism mobilization in order to adapt to an adverse situation (Selye, 1975). We observed significantly elevated levels of corticosterone 35 min after stress that were similar in all groups(Figures 2A, 3A, 4A, 5A). However, there was also a significant difference in the control condition of this comparison in the adult male group (Figure 5A). It was also expected that there would be a significant difference in the 35 min time point between the stress and control groups, but this was only the case for adolescent females (Figure 2A). These unexpected results may be due to the elevated plus maze being inherently stressful, and, as blood was collected after behavior scoring, may have elevated serum corticosterone beyond baseline even in the control animals. One week after stress, corticosterone concentration returned to levels similar to baseline in all groups, regardless of the treatment (Figures 2A, 3A, 4A, 5A), which indicates that the stressor does not result in chronic dysregulation of the HPA axis. HPA axis activation starts with the release of CRF by the periventricular nucleus of the hypothalamus. While it is known that there is a CRF-containing projection from hypothalamus to LC along with receptors in the LC that respond to glucocorticoids such as corticosterone (Wang et al., 2015), it is unclear if the changes in LC physiology we identified in adolescent males and females relate directly to activation of the HPA axis. This may be due to the fact that we did not detect a direct relationship between serum corticosterone levels and LC firing rates and were only able to find correlations between HPA axis activation and behavior, including parameters of both motor behavior and anxiety-like behavior, in adolescent males (Figure 6). The lack of a similar correlation in adult males may have to due with greater behavioral resilience to stress in adults than adolescents. Importantly, it has been shown that a genetic deletion of the glucocorticoid receptors in LC-NE neurons reduces social behavior in female but not male mice (Jacobson, 2019), which could explain why a similar HPA axis response leads to different behaviors. Although the HPA axis and LC/NE systems are both activated during stress, observations from this study suggest that they may operate independently of one another and not necessarily in a sequential or ordered fashion, despite the known role of CRF in LC activation. It is also interesting to note that, although the hormonal response to stress is similar in in different age groups, the adolescent LC and behavior are particularly susceptible to stress. Furthermore, the electrophysiological and behavioral adaptations among adolescent males and females are sex-specific: adolescent female rats showed a significant stress-induced increase in time spent in the center of the OFT and a decrease in LC neuronal excitability, while males showed opposite changes. It is difficult to assess whether the distinct behavioral changes in the OFT correspond directly to a stress-induced pro-exploratory and/or anxiolytic effect in females, or if anxiety-like behavior in the OFT manifests differently in males and females. It is interesting, however, that these behavioral changes occurred in parallel with opposing physiological changes within LC. This is notable because of the well-established relationship between LC discharge rates and behavioral indices of arousal. As such, it can at least be postulated that stressor exposure produces an anxiolytic effect, or perhaps reduces fear in response to a novel environment, in adolescent females one week later. It is also worth noting that both stressed adolescent and adult males are less active in the EPM, while only the adolescent male group continues this trend by being less active in the OFT at the one week time point.

These data also match well with findings from other studies which have shown that adolescent female and male rats differ in their stress response. For example, both male and female Wistar rats display reduced anxiety-like behavior in adulthood after acute lipopolysaccharide-induced inflammatory stress during adolescence, but acute and chronic restraint stress during adolescence reduced anxiety-like behavior only in females (Traslavina et al., 2014). Adolescent female Long-Evans also show decreased anxiety-like behavior after social stress without any effect on males (Bingham et al., 2011). On the other hand, it has been reported that peripubertal stress leads to increased risk-taking behavior and novelty seeking in Wistar Han male and female rats with more pronounced effect in females (Toledo-Rodriguez and Sandi, 2011). Our findings from adolescent female and male rats are also consistent with another recent publication (Lovelock and Deak, 2019), which used acute footshock stress during adolescence (30 PND) and reported long-term increased anxiety-like behavior in males but decreased anxiety-like behavior in females in adulthood (70 PND). One limitation of this study is that sexual maturity, signifying the beginning of adolescence, seems to occur earlier in females (P32–P34) than in males (P45–P48) (Sengupta, 2013). Therefore, the use of age-matched groups that fall in the range of adolescence for females but before adolescence in males may hinder any comparisons between groups.

The fact that there are disparities within and between male and female rats with regards to how they respond to stress may be explained in part by the variable nature and the duration of stressors used in the literature. Furthermore, while the behavioral effects of stress in both sexes have been extensively explored, there are few studies of sex differences in stress responsiveness of the LC/NE system in adolescent rats, despite its sexually dimorphic nature and a clear role in mediating arousal, vigilance, and anxiety-like behavior. Therefore, we assessed electrophysiological characteristics of LC neurons using whole-cell patch clamp electrophysiology. We found that, in contrast to LC neurons from adolescent male rats, which become more excitable following stressor exposure, those from stressed adolescent females are less excitable in response to current injection compared to control LC neurons. This was a surprising finding because the female LC/NE system is known to be more susceptible to CRF and has been demonstrated to be more sensitive to both acute hypotensive and swim stress (Curtis et al., 2006). Such decreased excitability of female LC neurons may be due to possible long-term effects of CRF signaling which alters gene expression and changes in ion channel trafficking (Borodovitsyna et al., 2018b). Additionally, a recent genetic analysis (Mulvey et al., 2018) demonstrated sex-differential expression of multiple genes in LC including elevated expression of the gene for the prostaglandin EP3 receptor in female mice. The same study demonstrated that activation of EP3 with the agonist sulprostone led to decreased anxiety-like behavior in female mice after restraint stress and decreased firing in female LC neurons compared to males. Similar mechanisms might contribute to the long-term effects observed in our study. However, this explanation cannot account for the apparent age-related difference between adolescent and adult females. Therefore, there must be some other mechanism for this difference, such as differences in circulating ovarian hormones due to age.

Another relevant question that this study raises is why adolescent male and female rats show opposing changes in anxiety-like behavior and LC neuronal physiology in response to stress, while human females are generally more susceptible to stressor exposure and have almost twice as high of an incidence of psychiatric conditions such as PTSD (Kessler et al., 1995), anxiety disorders (Bandelow and Michaelis, 2015), and depressive disorders (Angst et al., 2002). It has been demonstrated that repeated restraint stress decreases basolateral amygdala (BLA) neuronal firing and causes neuronal atrophy in the lateral nucleus of amygdala in females, while males show opposite changes in morphology and physiology (Blume et al., 2019). These data parallel our findings, and sex-specific changes in amygdala function may contribute to the sex-specific stress-induced changes in LC physiology we have identified in adolescent rats. As noted above, it remains to be determined if the increased time in the center of the OFT we identified in stressed females is an anxiolytic effect, or if coping strategies differ between males and females. This is an important question to consider because, despite conventional interpretation of increased center time in the open field as being indicative of reduced anxiety-like behavior, in humans posttraumatic dissociation syndrome and peritraumatic distress is described more frequently among female patients (Demarble et al., 2020). Therefore, impaired BLA function in response to repeated stress that has been reported in female rats, combined with the disrupted LC neuronal activity we observed in response to acute stress in female rats, might contribute to poor behavioral long-term adaptations in response to stress and higher risk of stress-associated psychiatric disorders in females. The amygdala is a major source of CRF for LC, and this pathway is responsible for LC activation and anxiety-like behavior formation during stress (McCall et al., 2015). A positive feedback activation loop was recently described from LC to BLA that contributes to anxiety-like behavior (McCall et al., 2017), indicating tight reciprocal control between these structures. Therefore, sexually dimorphic stress responses in one region may compound sexually dimorphic changes in the other.

While we observed opposing stress-induced changes in LC physiology and anxiety-like behavior between adolescent male and female rats, any such changes in adult animals regardless of sex were notably absent. This is of particular interest because in humans, adolescence is a critical period of development when many psychiatric disorders first emerge due to structural and functional synaptic changes in the CNS (Paus et al., 2008). Our data confirm that susceptibility of the adolescent LC/NE system to stress may contribute to the development of aberrant anxiety-like behavior. This is in contrast to the developed brain and LC/NE system observed in adults. Therefore, it is possible that, during the transition from adolescence to adulthood, adaptations may occur in LC and within LC targets which confer resilience to stressor exposure at both the behavioral and physiological levels. One reason for this could be due to changes in β-adrenergic receptor expression within the brain that promote emotional memory (Murchison et al., 2004; Schutsky et al., 2011). Notably, within the lateral amygdala, it has been shown that β-adrenergic receptors enhance neuronal firing in adolescent, but not adult, mice (Fink and LeDoux, 2018). It is also notable that in our study, stressor exposure resulted in increased excitability as measured by elevated action potential generation in response to current injection in adolescent males. Adult males, which did not show increased anxiety-like behavior following stressor exposure, also showed increased LC neuronal excitability, but as measured by elevated input resistance relative to controls. These parallel changes in LC neuronal excitability that manifest differently in adolescent and adult males may reflect unique stress-induced changes in channel physiology and function within each age group. For example, the increased action potential generation in the stressed adolescent males may be indicative of increased expression or function of voltage-gated sodium channels responsible for action potential generation. On the other hand, in adult males, which showed increased responsiveness to hyperpolarizing currents, but not increased action potential frequency in response to depolarizing, the change may be related to the closure of G-protein coupled inwardly-rectifying potassium channels that aid in determining resting membrane potential and resistance. It is also worth nothing that adolescent females showed opposite changes in LC action potential generation following stress relative to adolescent males, indicating that channel physiology and function in LC is dependent on stress history, age, and sex.

While this study investigated stress-induced changes in LC physiology and anxiety-like behavior occurred within groups of rats of specific ages and sexes, it did not explore any such differences between groups. This was done in order to preserve statistical power to detect effects within groups; however, others (Olpe and Steinmann, 1982) have reported that there is an age-related decline in LC firing rate. Therefore, the normal developmental trajectory for LC neurons as they progress from and adolescence to adulthood may represent a critical period when this development could be perturbed by stress. Our results are also consistent with findings from recent *in vivo* electrophysiology recordings after social stress (Zitnik et al., 2016), where adolescent male rats had elevated spontaneous firing of LC neurons while adult rats showed decreased firing. Another study similarly showed that social stress in early adolescence significantly increases LC spontaneous discharge relative to adult rats, and adult rats did not differ in LC tonic firing properties in response to social stress (Bingham et al., 2011).

The findings from these studies indicate that behavioral and LC neuronal responses to stressor exposure differ according to age and sex. These findings highlight the necessity to consider age and sex as important sources of variation when designing and performing experiments which investigate stress, behavior, and LC physiology. There are multiple factors involved in the development of psychiatric disorders such as anxiety disorders or PTSD, which include different brain areas and neurotransmitter systems, genetic predispositions and genetic polymorphisms, and emotional and behavioral coping strategies. While LC is only one of many systems that contributes to behavioral state and the stress response, these findings help to clarify how it contributes to these phenomena among rats of different age and sex. These disparities may further allude to important differences between male and female humans of different ages in the prevalence, progression, and pathophysiology of various clinical psychiatric conditions.

## Data Availability Statement

The raw data supporting the conclusions of this article will be made available by the authors, without undue reservation.

## Ethics Statement

The animal study was reviewed and approved by Rowan University SOM IACUC.

## Author Contributions

OB and JT: conceptualization, data curation, formal analysis, investigation, methodology, project administration, supervision, validation, visualization, roles/writing—original draft, and writing—review and editing. CC: data curation, formal analysis, investigation, methodology, validation, visualization. JL: conceptualization, project administration, and supervision. DC: conceptualization, funding acquisition, project administration, resources, supervision, validation, and writing—review and editing. All authors contributed to the article and approved the submitted version.

## Conflict of Interest

The authors declare that the research was conducted in the absence of any commercial or financial relationships that could be construed as a potential conflict of interest.

## Publisher’s Note

All claims expressed in this article are solely those of the authors and do not necessarily represent those of their affiliated organizations, or those of the publisher, the editors and the reviewers. Any product that may be evaluated in this article, or claim that may be made by its manufacturer, is not guaranteed or endorsed by the publisher.

## Abbreviations

LC: locus coeruleus
HPA axis: hypothalamic pituitary adrenal axis
CNS: central nervous system
PFC: prefrontal cortex
LC/NE: locus coeruleus/norepinephrine
CRFR1: corticotropin releasing factor receptor 1
CRF: corticotropin releasing factor
PND: postnatal days
OFT: open field test
EPM: elevated plus maze
aCSF: artificial cerebrospinal fluid
BLA: basolateral amygdala
PTSD: post-traumatic stress disorder
TMT: 2,4,5-trimethylthiazole
CI: confidence interval
SD: standard deviation
SEM: standard error of the mean.
